# Analgesic Efficacy and Safety of Local Infiltration Following Lumbar Decompression Surgery: A Systematic Review of Randomized Controlled Trials

**DOI:** 10.3390/jcm10245936

**Published:** 2021-12-17

**Authors:** Georgia Tsaousi, Parmenion P. Tsitsopoulos, Chryssa Pourzitaki, Eleftheria Palaska, Rafael Badenes, Federico Bilotta

**Affiliations:** 1 Department of Anesthesiology and ICU, School of Medicine, Aristotle University of Thessaloniki, University Campus, 54124 Thessaloniki, Greece; tsaousig@otenet.gr (G.T.); eleftheriapalaska@gmail.com (E.P.); 2 Department of Neurosurgery, Hippokration General Hospital, School of Medicine, Aristotle University of Thessaloniki, 54642 Thessaloniki, Greece; ptsitsopoulos@auth.gr; 3 Laboratory of Clinical Pharmacology, School of Medicine, Aristotle University of Thessaloniki, University Campus, 54124 Thessaloniki, Greece; chpour@auth.gr; 4 Department of Anaesthesiology and Intensive Care, Hospital Clìnico Universitario de Valencia, University of Valencia, 46010 Valencia, Spain; 5 Department of Anesthesiology and Critical Care, Policlinico Umberto I, “Sapienza” University of Rome, 00161 Rome, Italy; bilotta@tiscali.it

**Keywords:** lumbar spine surgery, discectomy, laminectomy, dexmedetomidine, magnesium, tramadol, steroids, wound infiltration, analgesia

## Abstract

This systematic review aims to appraise available clinical evidence on the efficacy and safety of wound infiltration with adjuvants to local anesthetics (LAs) for pain control after lumbar spine surgery. A database search was conducted to identify randomized controlled trials (RCTs) pertinent to wound infiltration with analgesics or miscellaneous drugs adjunctive to LAs compared with sole LAs or placebo. The outcomes of interest were postoperative rescue analgesic consumption, pain intensity, time to first analgesic request, and the occurrence of adverse events. Twelve double-blind RCTs enrolling 925 patients were selected for qualitative analysis. Most studies were of moderate-to-good methodological quality. Dexmedetomidine reduced analgesic requirements and pain intensity within 24 h postoperatively, while prolonged pain relief was reported by one RCT involving adjunctive clonidine. Data on local magnesium seem promising yet difficult to interpret. No clear analgesic superiority could be attributed to steroids. Τramadol co-infiltration was equally effective as sole tramadol but superior to LAs. No serious adverse events were reported. Due to methodological inconsistencies and lack of robust data, no definite conclusions could be drawn on the analgesic effect of local infiltrates in patients undergoing lumbar surgery. The probable positive analgesic efficacy of adjunctive dexmedetomidine and magnesium needs further evaluation.

## 1. Introduction

The presence of moderate to intense pain is typical after lumbar spine surgery, which in turn hampers early ambulation, recovery, and rehabilitation [1,2]. Most importantly, persistent pain might have serious consequences on patients’ quality of life [1,3]. This has produced a growing body of evidence to assess the safety and efficacy of traditional and novel analgesic modalities as effective means to alleviate disabling pain in patients subjected to lumbar spine surgery [4,5,6].

Nevertheless, due to the fact that each analgesic method presents certain benefits and drawbacks, the ideal analgesic regimen has yet to be determined.

Aiming to achieve an additive or synergistic analgesic effect by targeting different receptors in the peripheral and central pain signaling pathways [3,7], local infiltrative analgesia applied in layers of the surgical wound layers has been suggested as an appealing alternative due to its simplicity, enhanced safety, and limited cost [8,9]. A recent systematic review highlighted the clinical benefit of local anesthetic infiltration at wound closure following lumbar spine surgery by means of early pain perception, post-operative opiate requirements, and time to first analgesia request [4]. Nonetheless, the major concern remains the restricted duration of action from the use of local anesthetics as sole analgesic medications. To address this issue, alternative agents, namely opioids, non-steroidal anti-inflammatory drugs (NSAIDs), a-2 agents, opioids, steroids, ketamine, or magnesium, have been incorporated in the process of wound infiltration to enhance postoperative pain control [5]. 

However, the clinical advantage of the use of adjunct drugs in local wound infiltration during lumbar surgery has not yet been conclusively proven [4,5]. Therefore, the present systematic review aimed to appraise available clinical evidence on the efficacy and safety of wound infiltration with analgesics or miscellaneous drugs applied alone or in conjunction with local anesthetics in patients undergoing lumbar spine surgery concerning key outcome measures, namely postoperative pain intensity, supplemental analgesics consumption, time to first analgesic request, and adverse effects. 

## 2. Methods

### 2.1. Search Strategy 

A qualitative systematic review of the literature was conducted following the PRISMA (Preferred Reporting Items for Systematic Reviews and Meta-Analyses) methodology [10]. 

A dedicated study protocol was prepared at the commencement of the review and registered in the PROSPERO database (CRD 42021260829). 

According to the electronic literature search strategy, PubMed, Scopus, Embase, Cochrane Central Register of Controlled Trials (CENTRAL), and Web of Science databases were searched from their inception to 15 October 2021 to retrieve all randomized controlled trials (RCTs) pertinent to the implementation of wound infiltration using various pharmacologic agents applied either solely or as adjuncts to local anesthetics for postoperative pain control in lumbar spine surgery.

The medical subject headings MeSH terms “spine surgery”, “discectomy”, “laminectomy”, “local anesthetics”, “infiltration”, “pain”, “analgesia”, “analgesics”, “opioids”, “ketamine”, “tramadol”, “dexmedetomidine”, “clonidine”, “steroids”, “NSAIDs”, “non-steroidal anti-inflammatory drug”, “COX-2”, or “magnesium” with “AND” and “OR” as Boolean terms were applied into the databases to retrieve articles relevant to the objectives of the current review. An ultimate check of all databases was carried out on 2 November 2021. 

### 2.2. Study Selection 

Articles that were considered eligible for inclusion in this systematic review should fulfil the following criteria: (1) original RCTs comparing analgesics or miscellaneous drugs infiltrated over the skin line incision, subcutaneous and cutaneous tissues, and paravertebral muscles of both sides of the surgical wound [4], as adjuvants to local anesthetics (experimental intervention group) with active comparator (local anesthetic alone or combined to another agent) and/or placebo; (2) spine surgery involving discectomies and/or laminectomies; (3) adult population (age ≥ 18 years); (4) single-shot local wound infiltration was performed before or after operation; (5) complete data are available about the combined analgesic effect on total analgesic requirements and postoperative pain intensity assessment; and (6) full-text publications in the English language. 

Studies not fulfilling the aforementioned criteria as well as those testing wound infiltration with mixtures of various adjuncts or when only epinephrine was used as an adjunct (its applicability in wound infiltration causes vasoconstriction and prolongs the action of local anesthetics) were excluded from the final analysis. 

In compliance with the selection strategy, a stepwise selection process was followed by two reviewers (G.T. and P.P.T.) who independently screened and assessed the titles and the abstracts of the retrieved articles to select potential candidates for inclusion in this review. Duplicates or irrelevant records were discarded.

Full-text retrieval was reserved for those articles that their eligibility concerning the appropriateness of the research questions tested could not be ascertained from the title or abstract.

Any disagreement over eligibility was resolved by discussion. If a consensus could not be reached, an area expert (F.B.) blinded to the preceding estimations of the two reviewers was asked to resolve the dispute. Reference lists of recovered articles and related reviews were further scrutinized by another reviewer (E.P.) in an extra attempt to trace potentially relevant publications. 

### 2.3. Data Extraction and Outcome Measures

A dedicated data extraction form was developed to record all relevant data, involving publication details (author, year of publication), study arms (type of intervention and dosage), type of surgical procedure, anesthetic protocol, basic and rescue analgesic regimen, length of follow-up, and findings related to primary or secondary outcomes of interest.

The primary outcome measure was the effect of the applied intervention on total rescue analgesic consumption. The secondary outcomes included postoperative pain intensity as assessed by pain evaluation scores and time to first rescue analgesic administration after surgery completion and the occurrence of any local or systemic adverse events following wound infiltration. No pre-specified time points were applied for the outcomes. The studies were grouped according to the infiltrated adjunct to facilitate more clinically meaningful observations. 

### 2.4. Quality and Risk of Bias Assessment

Study quality was determined using the modified Jadad scale [11], which provides a low- to high-quality rating (from zero to 8) based on a summative score of eight items, namely randomization (maximum 2 points), blinding (maximum 2 points), withdrawals/dropouts (maximum 1 point), inclusion/exclusion criteria (maximum 1 point), adverse effects (maximum 1 point), and statistical analysis (maximum 1 point). Furthermore, selected RCTs were critically appraised by the Cochrane Collaboration Risk of Bias Tool [12], which incorporates the following domains: generation of the allocation sequence, allocation concealment, blinding of investigators and participants, blinding of outcome assessors, and incomplete outcome data. Each item was classified as a low, unclear, or high risk of bias. To minimize the impact of subjective interpretation, the methodological quality assigned to each trial was adjudicated by two reviewers (G.T., C.P.) independently. Any discrepancies were resolved by consensus. 

## 3. Results

### 3.1. Studies Selection

Initially, a total of 1540 potentially relevant records were retrieved from the database search, while three more were identified by hand search. The screening process was undertaken by two independent investigators (G.T. and P.P.T.). After removing all duplicates from the review of the titles and abstracts, 19 citations were selected for full-text analysis. Among them, EIGHT articles were discarded due to methodological constraints, not involving RCT, or lacking English full-text publication, leaving 11 studies for inclusion in the final qualitative appraisal. All selected reports were double-blind RCTs, enrolling a total of 868 adult patients.

**Figure 1:**
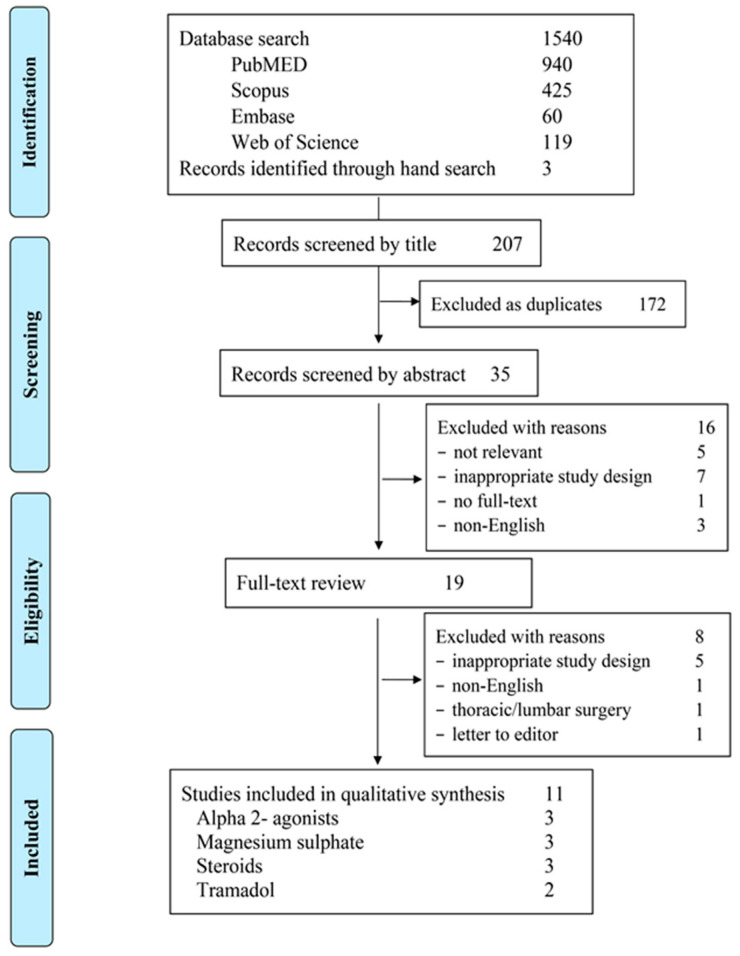
Flow diagram showing the results of the search and reasons for exclusion of studies.

Given the methodological and clinical heterogeneity among the included studies with regards to the type of surgery, the adjuncts studied, local anesthetics used, drugs dosage and volumes, as well as the applied baseline analgesic protocol, quantitative data analysis was not feasible. The findings from the literature review and study selection process are summarized in Figure 1.

### 3.2. Quality Assessment and Risk-of-Bias Estimation

The methodological quality of the included studies could be characterized as “satisfactory”, considering that the majority (*n* = 8) of the enrolled studies were graded with a score equal to or higher than 6 (Table 1). Likewise, the risk of bias was classified as “low to moderate” in eight out of 11 RCTs, with allocation concealment and incomplete prespecified outcomes reporting contributing most to the highest risk of bias (Table 2).

### 3.3. Description of Included Trials

The majority of the included RCTs implemented a two- [13,14,15,16,17,18] or three-arm [19,20] study design, whilst three RTCs incorporated either four [21] or five [22,23] comparison groups. It should be noted that one study tested four pharmacological regimens besides placebo; yet, they applied two identical groups assessed separately before and after surgery.

On all occasions, the experimental study group involved the adjunct co-infiltration with a local anesthetic compared either with an active comparator, namely the same adjunct plus an alternative local anesthetic [16,17,20], same local anesthetic plus an alternative adjunct [19], local anesthetic alone [13,14,15,21,22,23], and local adjunct infiltration (tramadol) [21] or placebo [21,22,23].

The tested adjuncts involved a2-agonists (clonidine [13], dexmedetomidine [14,15]), magnesium [16,17,18], steroids (methylprednisolone) [20,22,23], or tramadol [21]). A single study assessed the comparable analgesic efficacy of co-infiltration of two alternative adjuncts (tramadol or dexmedetomidine) with local anesthetic versus local anesthetic infiltration alone [19]. Of note, among the various types of lumbar spine surgeries being assessed in the study by Abdel-Hay et al. [13], only data from discectomies and laminectomies were evaluated.

Application of wound infiltration at the end of surgery was adopted by the majority of study designs, except for four RCTs, where this practice was implemented before incision [13,16] or incorporated pre- and post-incisional wound infiltration in a single study protocol [23,24]. It should be noted that the administration of an analgesic regimen upon surgery completion in addition to the basic analgesic protocol was defined in only two study protocols [13,14]. Specifically, Abdel Hay et al. [13] injected paracetamol (15 mg/kg) plus ketoprofen (1 mg/kg) after wound closure, while Daiki et al. [14] administered the same dose of paracetamol supplemented by morphine (0.1 mg/kg) and nefopam (20 mg).

All included studies provided a maximum of a 24-h follow-up period for postoperative pain assessment except for one report where the follow-up period extended to day 3 postoperatively. The basic characteristics of the reviewed studies and wound infiltration-related outcomes are shown in Table 1.

### 3.4. Analgesic Efficacy

Postoperative analgesics consumption, pain intensity assessment, and time to first analgesic demand constituted the combined end-points for the assessment of analgesic efficacy in the majority of the included studies [13,15,17].

#### 3.4.1. Alpha 2-Agonists

Three high-quality RCTs studied a2-agonists as adjuncts to local anesthetics for wound infiltration in lumbar spine surgery [13,14,15]. The co-infiltration of dexmedetomidine with ropivacaine before wound closure presented a clear analgesic superiority over ropivacaine infiltration since a notable reduction of analgesic demands and improvement of pain intensity scores was noted up to 24 h postoperatively [14,15], an effect further supported by at least two-fold prolongation of time to first rescue analgesic request [14,15]. However, it should be noted that the analgesic potency of pre-incisional adjunctive dexmedetomidine lasted shorter (up to 16 h) postoperatively. 

No dose-response relationship was seen, as the analgesic effect induced by 0.5 mcg/kg dexmedetomidine infiltration [14] was equivalent to that of 1 mcg/kg [15]. Similarly, the preemptive field infiltration with a mixture of clonidine 150 mcg and bupivacaine 0.25% (19 mL) reduced both postoperative rescue morphine requirements (up to day 3), and pain scores (up to day 8), compared with local anesthetic alone [13]. Of note, the enhanced quality of postoperative analgesia induced by the mixture of clonidine with bupivacaine was valid in both lumbar discectomies and laminectomies [13].

#### 3.4.2. Magnesium

Three moderate-quality RCTs assessed the analgesic potency of adjunctive magnesium 500 mg to local anesthetic applied for field infiltration after lumbar laminectomy [16,17,18]. The supplementation of bupivacaine with magnesium reduced postoperative tramadol use by 85–180 mg in 24 h and prolonged analgesic duration by 1.5 times, yet the pain intensity reduction was short-lived (up to 7.8 h postoperatively) [18]. However, there is contradictory evidence regarding the superiority of the mixture of magnesium with either bupivacaine or ropivacaine, as one RCT attributed higher analgesic quality to the combination of magnesium with bupivacaine [16], whereas another displayed the opposite effect [19].

#### 3.4.3. Steroids

Three moderate-to low-quality RCTs studied the use of methylprednisolone 40 mg as an infiltration adjunct in lumbar discectomy [22,23] or laminectomy [21]. No difference could be documented on the consumed amount of analgesics, pain intensity, and time to first rescue analgesic demand when wound infiltration with the combination of methylprednisolone plus a local anesthetic (bupivacaine [23] or levobupivacaine [20,22]) was compared to local anesthetic alone irrespective of the local anesthetic used [20]. Nonetheless, all groups induced an enhanced postoperative analgesic effect compared to placebo. However, when preemptive infiltration of active comparators was tested against their preclosure application in terms of pain-intensity control or prolongation of the interval to rescue analgesics request, no clear-cut superiority occurred. 

#### 3.4.4. Tramadol

Two high-quality RCT examined the analgesic efficacy of local anesthetic supplemented by tramadol 2 mg/kg to either active comparators alone (tramadol or local anesthetic) or placebo applied for wound infiltration in lumbar discectomy. No superiority could be demonstrated in the tramadol co-infiltration group, as its impact on rescue analgesic consumption or nociception ranged from insignificant [19] to notable reduction [21]. Furthermore, tramadol infiltration alone was less effective than tramadol co-infiltration with local anesthetic but superior to levobupivacaine alone or placebo [19]. Of note, the analgesic potency of adding dexmedetomidine to local anesthetic was two-fold stronger than tramadol co-infiltration alone [19]. 

### 3.5. Other Effects

The mean time to mobilization was shorter when adjunctive dexmedetomidine compared to local anesthetic alone was infiltrated to the wound (22 ± 3 and 27 ± 6 h, respectively; *p* < 0.001) [14]. Accordingly, the level of patients’ satisfaction was higher in cases of magnesium co-infiltration [18]. 

The occurrence of postoperative nausea and vomiting (PONV) constituted the single reported adverse event from the majority (9 out of 12) of the included RCTs. A limited number of studies recorded a beneficial effect of adjunctive dexmedetomidine [15], steroids [22,23], and tramadol [21] on PONV occurrence, whilst most studies failed to identify any profound difference between experimental groups and active comparators or placebo. There are insufficient data on other adverse effects, such as hemodynamic alterations, respiratory depression, agitation, sedation, dizziness, wound healing problems or infection, allergic reactions, itching, or urinary or bowel problems (Table 3).

## 4. Discussion

Our analysis indicates that the use of dexmedetomidine as a local anesthetic adjuvant for wound infiltration exerts a positive impact on analgesic requirements and pain intensity up to 24 h after lumbar spine surgery. The role of magnesium seems promising, yet the degree of its analgesic effect is less clear due to limited evidence. On the contrary, no clear-cut analgesic superiority can be acknowledged to steroids or tramadol applied as an add-on to local anesthetics. Of note, the impact of adjunctive dexmedetomidine, magnesium, steroids, or tramadol on PONV and the occurrence of other adverse events remains inconclusive.

Optimal analgesia after lumbar spine surgery should balance between the procedure-related burden of pain or analgesic-induced adverse events and delayed mobilization, further implicating the quality of recovery. 

Systemic opioids supplemented by nonsteroidal anti-inflammatory agents (NSAIDs) and/or paracetamol have been widely used for pain control after lumbar spine surgery. Nonetheless, the undisputed analgesic superiority of systemic opioids is frequently achieved at the expense of an enhanced incidence of respiratory depression, drowsiness, or PONV [24,25]. Although the intrathecal use of opioids has been demonstrated to provide satisfactory pain control in lumbar spine surgery, the potential side effects of this practice are an issue of major concern [4]. Similarly, NSAIDs may be implicated in coagulopathy disorders although they confer the benefit of vomiting reduction [26]. In this context, local infiltration of the surgical site with various medications has gained popularity over the established analgesic strategies to minimize systemic side effects while ensuring satisfactory pain relief [26]. Of note, the continuous wound infusion (CWI) via a catheter has been suggested as an alternative to the single-shot wound infiltration technique for various types of surgical procedures, yet CWI incurs a potential risk of systemic toxicity due to high local anesthetic plasma concentrations [9]. Pain following spine surgery is mediated through various neurophysiological and chemical pathways, including neuropathic, inflammatory, and nociceptive pain responses [27]. Surgical tissue injury activates an inflammatory cascade, further promoting the sensitization of peripheral nociceptors. This effect mediates an acute pain response known as primary hyperalgesia that eventually leads to exaggeration and protraction of postoperative pain [28,29,30]. The underlying peripheral mechanisms of most adjuvants used for wound infiltration analgesia have not been fully elucidated, yet several involved pathways have been proposed. 

### 4.1. Alpha 2-Adrenergic Agonists

The analgesic efficacy of peripheral alpha 2-adrenergic agonists (clonidine or dexmedetomidine) could be assigned to the inhibition of potential propagation, further blocking the transmission of pain signals and exerting an a2 receptor-independent inhibitory effects on nerve fiber action potential as well as their potent anti-inflammatory effects [31,32,33]. Over and above, these agents induce alpha-1-mediated vasoconstriction around the site of the injection, which delays the absorption of the local anesthetic and enhances the activity of co-infiltrated local anesthetic [34].

Current evidence demonstrates that adjunctive dexmedetomidine used in incisional infiltration could reduce the rescue analgesia rate and analgesic consumption within 24 h after surgery. A concomitant prolongation of the time to first analgesic requirement by as long as 10 h and pain score reduction up to 48 h postoperatively compared to sole local anesthetic infiltration can also be achieved [32].

In agreement with previous reports, the present systematic review documented a clear analgesic superiority of either pre-incisional or before wound closure co-infiltration of dexmedetomidine with ropivacaine over ropivacaine infiltration, as reflected by the notable reduction in analgesic demands and improved pain intensity scores early postoperatively [14,15], an effect further supported by at least two-fold prolongation of time to first rescue analgesic request [14,15]. No dose-response relationship for the local use of dexmedetomidine could be identified by our qualitative review, a finding further supported by recent evidence indicating that doses of adjunctive dexmedetomidine in a range between 0.5 μg/kg and 5 μg/kg are equally effective for postoperative pain relief in abdominal, head and neck, and breast surgery [5]. On the contrary, a recent meta-analysis demonstrated that adding low-dose dexmedetomidine (≤1.0 μg/kg) to local anesthetics induced a superior postoperative analgesic effect than high-dose DEX (>1.0 μg/kg) [32]. Thus, future research should focus on the dose-response effect of dexmedetomidine.

Clonidine (1 to 3 μg/kg) co-infiltration with local anesthetics in abdominopelvic surgery and tympanoplasty promoted a reduction of opioid requirements (by up to half) and pain scores reduction while prolonging analgesia duration (twofold to threefold) [5]. In line with previous findings, our study identified a prolonged analgesic effect as a result of pre-incisional field infiltration with a mixture of clonidine and bupivacaine, reflected by the reduced rescue morphine requirements up to day 3 and pain scores up to day 8 [13]. It should be emphasized that among surgical subgroups, the discectomy subgroup benefited most from this combination. However, the clinical relevance of these findings remains unclear. 

Furthermore, the existing evidence on this topic supports the enhanced safety profile of dexmedetomidine [5,32], while a higher incidence of hemodynamic compromise and mild sedation has been reported by the use of higher doses of adjunctive clonidine infiltration, such as 250 μg [35,36].

### 4.2. Magnesium 

Although the use of systemic magnesium has been consistently reported enhancing the analgesic properties of other established analgesic agents in various acute pain states, the antinociceptive effects of wound infiltration with magnesium are less documented [1,37,38,39]. It has been speculated that magnesium sulfate infiltration modifies the magnitude of nociception primarily via non-competitive antagonism of peripheral N-methyl- aspartate (NMDA) receptors in the skin and muscles involved in the sensory transmission of noxious signals [40,41]. Over and above, the existence of NMDA receptors has been evidenced in the peripheral C fibers, the activation of which contributes to pain sensitization and hyperalgesia [40]. Another plausible mechanism responsible for the analgesic effects of magnesium could be the local activation of the endothelium-derived nitric oxide (EDNO) pathway. Accumulating evidence indicates that nitric oxide is directly involved in the nociceptive process inhibiting both peripheral and central transmission of noxious stimuli, an effect supplemented by nitric oxide-induced potentiation of the analgesic effect of opioids and other analgesic substances [42].

The reduced postoperative analgesic requirements, analgesia duration, and pain perception recorded in the present systematic review [16,17,18] could be attributed to the additive or synergistic interaction between local anesthetics and magnesium as a result of peripheral NMDA receptors antagonism and thus attenuation of pain impulse transmission [43]; this is an effect valid up to approximately 8 h postoperatively [16,18]. Of note, the magnitude of this favorable impact on postoperative pain relief was more pronounced in rescue analgesics consumption compared to other indices. This could be explained by the fact that the latter index is regarded as a more accurate predictor of analgesic efficacy compared to the time interval of the first analgesic request or pain perception. Possible reasons for the inconsistency regarding the analgesic potency of wound infiltration with the addition of magnesium to either ropivacaine or bupivacaine [16,17] could be the different dosing regimens of the infiltrated local anesthetics [44] or variances in the applied wound infiltration practices [45]. 

Current literature indicates that magnesium as an infiltration adjunct promotes an opioid-sparing effect yet produces minimal pain reduction [5]. Although magnesium infiltration does show some analgesic potential as an adjunct to co-infiltration, it is a cheap and relatively safe drug and, as such, is more likely to be cost-effective if its use is accompanied by a postoperative opioid-sparing effect; more evidence is needed to discern its real effect and value [5].

### 4.3. Corticosteroids

Although the exact mechanism of antinociception induced by local corticosteroids remains unidentified, it is thought to limit the release of inflammatory mediators, suppress neuronal discharge, or impede transmission in nociceptive C fibers when injected directly into the surgical wound. Nonetheless, co-infiltration of methylprednisolone 40 mg with local anesthetics in lumbar discectomies or laminectomies was no better than local anesthetic infiltration alone [20,22,23], which is a finding confirmatory of the existing evidence regarding methylprednisolone infiltration in knee arthroplasty [46]. The mixture of methylprednisolone plus local anesthetic achieved an enhanced analgesic potency compared only to placebo. Therefore, the evidence appears to counter the use of methylprednisolone as an infiltration adjunct. Notwithstanding, additive dexamethasone to LA infiltration presented a marginal analgesic benefit compared with LA alone in laparoscopic cholecystectomy [47] and cesarean section procedures [48]. In accordance with previous investigations [5], methylprednisolone co-infiltration was not associated with significant adverse effects, whist limited and mixed evidence indicates that this practice is advantageous regarding the postoperative occurrence of PONV versus placebo [20,22,23].

### 4.4. Tramadol

Locally infiltrated tramadol exerts a topical analgesic effect through a mechanism that has not yet been fully elucidated [26,49]. It has been suggested that tramadol-induced antinociception is mediated via an inhibitory effect on M1 and M3 muscarinic acetylcholine receptors and NMDA receptors in peripheral tissues and nerves while also promoting anti-inflammatory effects by reducing substance P and norepinephrine [50]. Moreover, tramadol, similar to Las, prolongs the opening of voltage-dependent K+ ion channels, further influencing the hyperpolarization of neurons [50,51].

The synergistic local anesthetic effect of tramadol on local anesthetic drugs was supported by a single RCT showing that the analgesic potency of the combination of tramadol and levobupivacaine infiltration at the end of lumbar discectomy provided better analgesic control in comparison to either drug alone [21]. Of interest, no analgesic benefit could be attributed to the co-infiltration of tramadol with ropivacaine compared with sole ropivacaine although an opioid-sparing effect of adjunctive dexmedetomidine to tramadol co-infiltration was documented [19].

A growing number of studies have assessed the impact of local tramadol on postoperative pain relief after non-cardiac surgeries [5]. Further reinforcing our findings, these studies demonstrated the clear analgesic superiority of tramadol over placebo though its co-infiltration with LA provided inconclusive findings, questioning its use in this context [5]. Although local tramadol has been suggested as a promising alternative to traditional opioids for postoperative pain relief as monotherapy due to its comparative lack of respiratory depression and sedative properties, evidence on its value in co-infiltration with LA is mixed and insufficient to support its benefit as an adjunct to LA infiltration [5]. Importantly, infiltrated tramadol at the dose studied did not increase the rate of adverse effects, presumably through sparing systemic opioid needs. Overall, given the sparse available data, the analgesic efficacy of local tramadol should be interpreted with caution 

### 4.5. Study Limitations

Several limitations should be acknowledged. Foremost, there were methodological discrepancies involving variability in the performed surgical procedures (discectomies, and/or laminectomies,), anesthetic and analgesic regimens, type and doses of local anesthetic, infiltration techniques, and timing of incisional infiltration among the reviewed studies. The limited available data prohibited further subgroup investigation and thus a thorough antinociception evaluation analysis. Considering that all but two [20,22] of the included trials compared multiple postoperative time points, intervention groups, or primary and secondary outcomes without performing any correction for multiple testing or multivariable analyses, the possibility of a type I error and the likelihood of attributing benefits to drug infiltration should be expected. Finally, in all but one of the included studies, the follow-up period was limited to the first postoperative day; hence, the long-term effects of adjunctive drugs infiltration remain unclear.

## 5. Conclusions

Available evidence suggests that critical appraisal of studies related to pain relief after lumbar spine surgery cannot provide any definite recommendations regarding pharmacological interventions adjunctive to local anesthetics for wound infiltration. Dexmedetomidine and magnesium show potential, but more evidence is needed to establish their clinical role as adjuncts to local anesthetic. On the contrary, incisional co-infiltration with methylprednisolone or tramadol failed to demonstrate any profound analgesic superiority over local anesthetics. None of the assessed adjuncts induced any serious adverse effects, but the magnitude of this effect was less clear due to non-systemic reporting of miscellaneous events. 

## 6. Implications for Future Research

Future adequately powered and rigorously designed trials should focus on the effectiveness and safety of these adjuncts, with particular emphasis on the most promising regimens. These studies should assess not only pain control or intervention-related complications but also outcome end-points, such as time to ambulation, quality of recovery, length of hospitalization, and the attenuation of chronic pain symptoms.

Finally, the efficacy of wound infiltration needs also to be assessed in chronic analgesic consumers as it is a phenomenon commonly encountered in this surgical population. 

## Figures and Tables

**Table 1:** Critical appraisal of randomized controlled trials assessing local wound infiltration for pain control after lumbar spine surgery using the modified Jadad score.

Author	Design	Jadad Score						
		Total	Randomization	Blinding	Attrition Info	Inclusion/ Exclusion Criteria	Adverse Effects Method	Statistical Analysis Info
Abdel Hay et al., 2017 [13]	Double-blind, RCT	7	2	2	1	1	0	1
Daiki et al., 2019 [14]	Double-blind, RCT	8	2	2	1	1	1	1
Deshwal et al., 2018 [15]	Double-blind, RCT	7	2	2	1	1	1	0
Hazarika et al., 2017 [16]	Double-blind, RCT	8	2	2	1	1	1	1
Sane et al., 2020 [17]	Double-blind, PBO-controlled, RCT	6	2	2	0	1	0	1
Donadi et al., 2014 [18]	Double-blind, RCT	6	2	1	0	1	1	1
Mitra et al., 2017 [19]	Double-blind, RCT	8	2	2	1	1	1	1
Gurbet et al., 2014 [20]	Double-blind, PBO-controlled, RCT	6	0	2	1	1	1	1
Ozyilmaz et al., 2012 [21]	Double-blind, RCT	5	0	2	0	1	1	1
Gurbet et al., 2008 [22]	Double-blind, PBO-controlled, RCT	5	1	1	0	1	1	1
Ersayli et al., 2006 [23]	Double-blind, PBO-controlled, RCT	4	0	1	0	1	1	1

Notes: RCT, randomized controlled trial; PBO, placebo.

**Table 2:** Critical appraisal of bias of the included trials assessing local wound infiltration for pain control after lumbar spine surgery using Cochrane Collaboration of Risk tool.

Author	Random Sequence Generation	Allocation Concealment	Personnel and Participants	Outcome Assessors Blinded	Incomplete Outcome Data	Selective Reporting	Other Bias	Final Estimation
Abdel Hay et al., 2017 [13]	Low	Unclear	Low	Low	Low	Low	High	Low
Daiki et al., 2019 [14]	Low	Unclear	Low	Low	Low	Low	Low	Low
Deshwal et al., 2018 [15]	Low	Low	Low	Low	Moderate	Moderate	Low	Low
Hazarika et al., 2017 [16]	Low	Unclear	Low	Low	High	Moderate	Low	Moderate
Sane et al., 2020 [17]	Low	Low	Low	Unclear	Moderate	Low	Moderate	Moderate
Donadi et al., 2014 [18]	Low	Unclear	Low	Unclear	High	Unclear	High	High
Mitra et al., 2017 [19]	Low	Unclear	Low	Low	Low	Low	Low	Low
Gurbet et al., 2014 [20]	High	Low	Low	Unclear	Moderate	High	Moderate	Moderate
Ozyilmaz et al., 2012 [21]	High	Low	Low	Low	Low	Low	Low	Low
Gurbet et al., 2008 [22]	High	High	High	Unclear	High	High	High	High
Ersayli et al., 2006 [23]	High	High	High	Unclear	Low	Low	High	High

**Table 3:** Characteristics of the included studies involving local wound infiltration for pain control after lumbar spine surgery.

Study ID	Study Arms	No Pts	Type of Surgery	Anesthesia Protocol	Basic Analgesia/Rescue Analgesia	Follow-Up	Primary Outcome	Secondary Outcomes		
							Analgesic Requirements	Pain Intensity	Time (h) to Rescue Analgesic	Other Effects
Abdel Hay et al., 2017 [13]	Bupi 0.25% 19 mL + CLON 150 μg 1 mL vs. Bupi 0.25% 20 mL Pre-incisional	225 (Bupi + CLON (116)/Bupi (109))	Laminectomy/Discectomy	PROP 3 mg/kg + SUF 10 mcg and SEVO (+N_2_O) + SUF 5 mcg boluses (iv)	APAP 1gr + Ketoprofen 50 mg (iv) every 6 h/Morphine 5 mg (sc) up to Day 3	NRS/2 h up to day 2 and /8 h from day 3 to day 8 and morphine up to day 3	Lower in Bupi + CLON vs. Bupi group (*p* < 0.001) in lumbar stenosis surgery	AUC of NRS lower in Bupi + CLON vs. Bupi group (*p* < 0.05) in all lumbar discectomy and stenosis surgery	N/A	Hemodynamics (0) Atelectasis (ns) Superficial wound infection (ns)
Daiki et al., 2019 [14]	Ropi 2 mg/kg + DEX 0.5 mcg/kg (30 mL) vs. Ropi 2 mg/kg (30 mL) End of surgery	63 (Ropi + DEX (33)/Ropi (30))	Discectomy	PROP 2.5 mg/kg + Remi 015 μg/kg and PRO (6 mg/kg/h) + Remi 0.05–2 mcg/h (iv)	APAP 1 gr or Tram or Ketoprofen 50 mg upon request (iv)	VAS at 0, 2, 4, 6, 12, 18, and 24 h and total analgesics up to 24 h	Lower in Ropi + DEX (median 0 mg) vs. Ropi (median 3 mg) in morphine equivalents (*p* < 0.001)	VAS lower in Ropi + DEX vs. Ropi group up to 24 h (*p* < 0.001)	Longer in Ropi + DEX (median 21 h) vs. Ropi (median 8 h) group (*p* < 0.001)	PONV (ns) Sedation (ns) HR higher in Ropi vs. Ropi + DEX (*p* = 0.002) MAP (ns) Urinary retention (ns)
Deshwal et al., 2018 [15]	Ropi 0.2% 30 mL + DEX 1 mcg/kg vs. Ropi 0.2% 30 mL End of surgery	60 (30 per group)	Discectomy	PROP 2 mg/kg + FNT 2 μg/kg and SEVO (+N_2_O) + FNT 1 mcg/h (iv)	PCA FNT 25 mcg/dose 4-h limit 400 mcg	VAS and PPS (static and dynamic) at 0, 0.5,1, 2,4,6,12, and 24 and FNT up to 24 h	Lower in Ropi + DEX (294 ± 39 mcg) vs. Ropi (470 ±3 0 mcg) group (*p* < 0.001)	VAS and PPS (dynamic) lower in Ropi + DEX vs. Ropi group up to 24 h (*p* < 0.001)	N/A	Hemodynamics (0) PONV (0) Wound infection (0)
Hazarika et al., 2017 [16]	Bupi 50 mg + Mg 500 mg (20 mL) vs. Ropi 50 mg + Mg 500 mg (20 mL) End of surgery	60 (Bupi + Mg(30)/Ropi + Mg (31))	Laminectomy	PROP 2 mg/kg + FNT 2 μg/kg and ISO + FNT 1 mcg/kg/h (iv)	Nalbuphine 5 mg/3 h on demand	VAS hourly up to 24 h	Lower in Bupi + Mg (12 ± 4) vs. Ropi + Mg (15 ± 5) (*p* < 0.01)	VAS lower in Bupi + Mg vs. Ropi + Mg from 4 h to 8 h	Longer in Bupi + Mg (7.3 ± 0.4) vs. Ropi + Mg (6.6 ± 0.7) (*p* < 0.001)	Agitation, enhanced hemodynamics in Bupi + Mg at 7 h and 8 h/Ropi + Mg at 6 h and 7 h Urinary retention (ns)
Sane et al., 2020 [17]	Ropi 70 mg + Mg 500 mg (20 mL) vs. Bupi 70 mg + Mg 500 mg (20 mL) End of surgery	60 (30 per group)	Laminectomy	PROP 2 mg/kg + FNT 1 mcg/kg (iv) and ISO + REMI 1 mc/kg/min	PCA morphine 2 mg/bolus	VAS at 6,12, and 24 h Analgesics up to 24 h	Lower in Ropi + Mg (mean 185 mg) vs. Bupi + Mg (mean 220 mg) groups (*p* = 0.03)	VAS lower at 6 and 12 h in Ropi + Mg (mean 2.8 and 2.9) vs. Bupi + Mg (mean 3.7 and 4) (*p* < 0.05)	N/A	Hemodynamics (ns)
Donadi et al., 2014 [18]	Bupi 0.25% 20 mL + Mg 500 mg vs. Bupi 0.25% 20 mL End of surgery	60 (30 per group)	Laminectomy	THIOP 4–7 mg/kg + FNT 2 mcg/kg and ISO+ FNT 1–5 mcg/kg/h (iv)	Tram 100–150 mg (im)	VAS at 0, 1, 2, 4, 8, 12, and 24 h Analgesics up to 24 h	Lower tramadol in Bupi + Mg (117 ± 63.4 mg) vs. Bupi (202 ± 76 mg) group (*p* < 0.0001)	VAS lower in Bupi + Mg vs. Bupi group up to 4 h (*p* < 0.05)	Longer in Bupi + Mg (7.8 ± 1.3 h) vs. Bupi (4.6 ± 0.9 h) group (*p* < 0.0001)	Satisfaction higher in Bupi + Mg (2.7 ± 0.6) vs. Bupi (2 ± 0.5) group (*p* < 0.001) PONV, urinary retention, dry mouth, allergic reactions, respiratory depression (0)
Gurbet et al., 2014 [20]	LevoBupi 0.25% 20 mL + MP 40 mg vs. Bupi 0.25% 20 mL + MP 40 mg vs. PBO End of surgery	60 (30 per group)	Laminectomy	PROP 3 mg/kg + FNT 2 μg/kg and SEVO (+N_2_O) + FNT boluses (iv)	PCA morphine 2 mg/bolus (iv) /Diclofenac 20 mg (im)	VAS (static and dynamic) up to 24 h/morphine up to 24 h	Lower in LevoBupi + MP (9.9 ± 2.1 mg) and Bupi+MP (9.4 ± 1.9 mg) vs. PBO (30 ± 5.6 mg) (*p* < 0.001)	VAS in LevoBupi + MP and Bupi + MP (ns) VAS lower in treatment groups vs. PBO up to 4 h (*p* < 0.001)	Longer in LevoBupi + MP (53 ± 16 min) and Bupi+MP (56 ± 17 min) vs. PBO (32 ± 14 min) (*p* < 0.001)	Sedation, nausea (ns)
Gurbet et al., 2008 [22]	LevoBupi 0.25% 30 mL + MP 40 mg vs. LevoBupi 0.25% 30 mL end of surgery vs. LevoBupi 0.25% 30 mL + MP 40 mg vs. LevoBupi 0.25% 30 mL (preemptive) vs. PBO End of surgery	80 (20 per group)	Discectomy	PROP 2–2.5 mg/kg + FNT 1–1.5 μg/kg and SEVO (+N_2_O) + FNT boluses (iv)	PCA morphine 2 mg/bolus and 4-h limit 0.4 mg/kg (iv)/Diclofenac 75 mg (im)	VAS at 1,4,8,16,20, and 24 h Analgesics up to 24 h	Similar in all tested groups vs. PBO (ns)	VAS lower in LevoBupi + MP and LevoBupi (end of surgery) vs. other tested groups (*p* < 0.05)	Longer in all tested groups vs. PBO (*p* < 0.05) Longer in LevoBupi + MP and LevoBupi (end of surgery) vs. LevoBupi + MP and LevoBupi (preemptive) (*p* < 0.01)	Sedation (ns) Nausea higher in PBO vs. other tested groups (*p* < 0.05) Vomiting (ns)
Ersayli et al., 2006 [23]	Bupi 0.25% 30 mL + MP 40 mg vs. Bupi 0.25% preemptive Bupi 0.25% 30 mL + MP 40 mg vs. Bupi 0.25% vs. PBO End of surgery	75 (15 per group)	Discectomy	PROP 2–2.5 mg/kg + FNT 1–1.5 μg/kg and SEVO (+N_2_O) + FNT boluses (iv)	PCA morphine 4-h limit 0.4 mg/kg (iv)	VAS and VER (static and dynamic) at 1, 4, 8, 16, 20, and 24 h and morphine up to 24 h	Lower in all tested groups vs. PBO (*p* < 0.001) Lower in preemptive Bupi + MP vs. other groups (*p* < 0.05)	VAS lower in preemptive Bupi + MP and Bupi groups vs. other groups up to 16 h (*p* < 0.05)	Longer in all tested groups vs. PBO (*p* < 0.05) Longer in preemptive Bupi + MP vs. other groups (*p* < 0.05)	PONV higher in PBO (*p* < 0.05) Sedation (ns)
Ozyilmaz et al., 2012 [21]	LevoBupi 0.5% 20 mL + Tram 2 mg/kg vs. Tram 2 mg/kg vs. LevoBupi 0.5% 20 mL vs. PBO End of surgery	80 (20 per group)	Discectomy	PROP 2 mg/kg + FNT 1 μg/kg and SEVO (+N_2_O) + FNT 50 mcg boluses (iv)	PCA pethidine 10 mg/bolus (iv)4-h limit 100 mg/Diclofenac 75 mg/12 h (iv)	VAS at 0, 1, 2, 4, 8, 12, and 24 h Analgesics up to 24 h	No patient in LevoBupi + Tram required analgesia Lower in Tram (37 ± 35 mg) vs. LevoBupi (129 ± 78 mg) vs. PBO (196 ± 71 mg) group (*p* < 0.001)	VAS lower in all tested groups vs. PBO up to 1 h (*p* < 0.001) VAS lower in LevoBupi + Tram and Tram vs. LevoBupi and PBO up to 4 h and 12 h (*p* < 0.05) VAS similar in LevoBupi and Tram up to 24 h (ns)	Longer in LevoBupi + Tram (803 ± 268 min) vs. LevoBupi (163 ± 216 min) vs. PBO (11 ± 2 min) group (*p* < 0.001)	PONV lower in LevoBupi + Tram group Itching (0)
Mitra et al., 2017 [19]	Ropi 0.5% 20 mL + Tram 2 mg/kg vs. Ropi 0.5% 20 mL + DEX 0.5 mcg/kg vs. Ropi 0.5% 20 mL End of surgery	45 (15 per group)	Discectomy	PROP 2 mg/kg + FNT 2 μg/kg and SEVO (+N_2_O) + FNT 1 mcg/kg boluses (iv)	Diclofenac 75 mg (im)	VAS at 0, 2, 4, 6, 12, 18, and 24 h Analgesics up to 24 h	Lower in Ropi + DEX (median 75 mg) vs. Ropi + Tram and Ropi (median 150 mg for both) groups (*p* = 0.008)	VAS lower in Ropi + DEX vs. Ropi group up to 24 h (*p* < 0.05)VAS lower in Ropi + DEX vs. Ropi + Tram group from 2 to 6 h (*p* < 0.01)	Longer median time in Ropi + DEX (930 min) vs. Ropi + Tram (420 min) and Ropi (270 min) group (*p* < 0.001)	Hemodynamics (ns) Sedation (ns) Nausea (ns)

Abbreviations: APAP, paracetamol; AUC, area under ROC curve; Bupi, Bupivacaine; CLON, Clonidine; DIC, Diclofenac; FNT, fentanyl; ISO, Isoflurane; MP, Methylprednisolone; NRS, Numerical Rating Scale; PBO, placebo; PONV, postoperative nausea and vomiting; PPS, Postoperative Pain Score; PROP, propofol; REMI, Remifentanil; SEVO, Sevoflurane; SUF, sufentanil; Tram, Tramadol; VAS, Visual Analogue Scale; VER, Verbal Analogue Scale; im, intramuscular; N/A, not assessed; ns, non-significant; h, hour; pts, patients.
